# Optimizing antidiabetic properties of *Galega officinalis* extract: Investigating the effects of foliar application of chitosan and salicylic acid

**DOI:** 10.1002/fsn3.4204

**Published:** 2024-05-27

**Authors:** Farinaz Angouti, Hassan Nourafcan, Sakineh Saeedi Sar, Assad Assadi, Raheleh Ebrahimi

**Affiliations:** ^1^ Department of Horticultural Science and Agronomy, Science and Research Branch Islamic Azad University Tehran Iran; ^2^ Department of Horticulture, Medicinal Plants and Organic Products Research Center, Miyaneh Branch Islamic Azad University Miyaneh Iran; ^3^ Department of Agricultural Science Technical and Vocational University (TVU) Tehran Iran; ^4^ Department of Veterinary Medicine, Miyaneh Branch Islamic Azad University Miyaneh Iran

**Keywords:** alkaloid, chitosan, diabetes, elicitor, *Galega officinalis*, metformin, salicylic acid, total phenolics

## Abstract

Diabetes poses a significant global health burden, demanding safe and effective therapeutic interventions. Medicinal plants offer promising avenues for natural diabetic management. *Galega officinalis* (goat's rue) has long been recognized for its hypoglycemic potential, but optimizing its phytochemical content and antidiabetic activity remains a key challenge. This study aimed to address this aspect by investigating the impact of foliar application of chitosan and salicylic acid on the physiological and phytochemical properties of *G. officinalis*, and subsequently evaluating its antidiabetic efficacy compared to that of the established drug metformin. A randomized complete block design with three replications was employed. Laboratory mice were divided into treatment groups receiving *G. officinalis* extract from plants sprayed with four salicylic acid concentrations (0.5–3 mM/L) and four chitosan concentrations (0–0.8 g/L). Blood glucose levels and various physiological parameters were assessed. Chitosan at 0.4 g/L and salicylic acid at 2 mM significantly enhanced the growth, photosynthetic pigments, and antioxidant activity of *G. officinalis*. Notably, the extract from plants treated with 3 mM salicylic acid exhibited the highest total alkaloid content, a potential contributor to antidiabetic activity. In a separate study, diabetic mice treated with this optimized *G. officinalis* extract (50 mg/kg) exhibited significantly greater blood glucose reductions compared to those treated with metformin (500 mg). This study demonstrates the potential of chitosan and salicylic acid in optimizing the beneficial properties of *G. officinalis*. The extract derived from plants treated with 3 mM salicylic acid displayed superior blood glucose‐lowering efficacy compared to metformin, suggesting its promising role as a potential natural antidiabetic therapy. Further research is warranted to elucidate the specific bioactive compounds responsible for this enhanced activity and translate these findings into clinical applications.

## Graphical abstract

The antidiabetic properties of *Galega officinalis* were enhanced by foliar application of chitosan and salicylic acid. Galega extract exhibited superior blood glucose‐lowering efficacy compared to metformin, suggesting its potential as a natural treatment for diabetes.

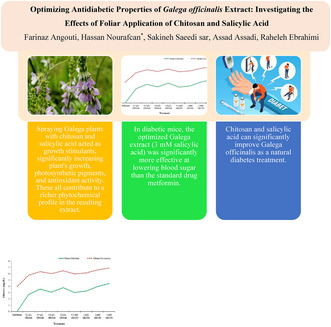

## 1 INTRODUCTION

Diabetes mellitus, characterized by chronic hyperglycemia, constitutes a global public health crisis, affecting over 422 million people worldwide. In Iran alone, an estimated 7.7 million individuals currently live with diabetes, with this number projected to rise significantly in the coming years. This alarming prevalence underscores the urgent need for effective and accessible therapeutic interventions. While mainstay medications like metformin offer glycemic control, concerns regarding long‐term side effects and potential drug resistance necessitate the exploration of complementary and alternative approaches (Lankatillake et al., 2019; Safari‐Faramani et al., 2019).

In this context, medicinal plants with established antidiabetic properties emerge as promising candidates.*Galega officinalis*, belonging to the Fabaceae family, has a long history of traditional use in managing diabetes, particularly in Eastern Europe and Russia. Its leaves and aerial parts contain bioactive compounds, including guanidine derivatives like galegine, exhibiting structural and functional similarities to the widely used antidiabetic drug metformin (Karakaş et al., 2016). However, optimizing the production and bioactivity of these beneficial phytochemicals within Galega remains a crucial challenge.

Elicitors – natural or synthetic compounds stimulating secondary metabolite production – offer a promising strategy for augmenting the therapeutic potential of medicinal plants (Ahmadi Moghadam et al., 2013).

Salicylic acid (SA), a naturally occurring phenolic compound, has demonstrated efficacy in inducing the biosynthesis of various health‐promoting metabolites in plants (Ali, 2021). Salicylic acid or ortho‐hydroxybenzoic acid is a phenolic compound that plays an important role in regulating plant physiological processes, such as germination, seedling growth (Shakirova et al., 2003), stomatal opening and closure, stomatal conductance (Raskin, 1992), membrane permeability (Barkosky & Einhellig, 1993), photosynthetic pigment composition (Hayat et al., 2005), flowering, phenolic compounds, ion absorption (Hayat et al., 2005), and respiration rate (Khan & Anderson, 2003). Foliar spraying of salicylic acid causes an increase in antioxidant capacity and carotenoids. It limits the rate of lipid peroxidation, reduces the amount of oxygenated water production while protecting cells, photosynthetic membranes, and photosynthetic pigments (Costa et al., 2005).

Similarly, chitosan, a biodegradable polysaccharide derived from chitin, has exhibited potential as an elicitor, enhancing plant defense mechanisms, growth parameters, and the accumulation of antioxidant compounds. Chitosan is reportedly used for coating seeds (Mahdavi et al., 2013) and fruits (Devlieghere et al., 2004; Uthairatanakij et al., 2007). Also, it is used as a fertilizer and an elicitor for controlling the release of toxic compounds (Sukwattanasinitt et al., 2001). It can stimulate the plant immune system, plant growth, and germination to protect plants against microbial attacks (Hadwiger et al., 2002). Chitosan has been reportedly used on basil (*Ocimum basilicum*) to increase total phenol content, flavonoids, anthocyanins, chlorophyll a and b, as well as carotenoids (Naderi et al., 2014). Chitosan reportedly increased total chlorophyll content, as well as chlorophyll a and b in beans (Sheikha & Al‐Malki, 2009). Chitosan increased phenolic compounds in white flax (*Linum album*) (Esmaeilzadeh‐Gharedaghi et al., 2012).

Diabetes mellitus (i.e., high blood sugar) is a metabolic disorder associated with the failure to produce insulin in the body. The body can become resistant to insulin, and, thus, insulin becomes less effective in its normal function. The main role of insulin is to lower blood sugar by different mechanisms. Disruptions in the function of insulin cause high levels of glucose in the blood and lead to disorders in carbohydrate, fat, and protein metabolism (Genuth et al., 2003). As the disease progresses, it causes damage to different tissues in the body, with a variety of side effects, including visual impairment (Duh et al., 2017), kidney disorders, cardiovascular problems, nerve dysfunctions, and wounds (Wallace et al., 2002). Thus, finding methods with fewer side effects to treat this disease is an important objective in this field of research.

Galega can be used therapeutically on diabetes (Nagalievska et al., 2018). Russia, Ukraine, and Belarus reportedly use Galega among the five most prominent medicinal plants for antidiabetic formulations (Hachkova et al., 2021). Given the individual potential of Galega, SA, and chitosan in promoting antidiabetic bioactives, the present study investigates their combined effect on Galega's phytochemical profile and antidiabetic activity. We hypothesize that foliar application of these elicitors can synergistically enhance the production of key bioactive compounds within Galega, leading to improved glycemic control efficacy compared to conventional therapeutic options.

## 2 MATERIALS AND METHODS

The seeds of Galega were provided by the Research Center for Natural Resources and Medicinal Plants of East Azarbaijan province. The seeds were planted in trays and transplanted to a field in the vicinity of Miyanah (longitude 47°72′, latitude 37°42′, and altitude 1082 m) in April 2019. Planting distances were 25 × 30 cm between the seedlings in the field. An experimental design of randomized complete blocks was arranged in nine treatments, including foliar spraying with four levels of salicylic acid (0.5, 1, 2, and 3 mM) and foliar spraying with four levels of chitosan (0.2, 0.4, 0.6, and 0.8 g/L). Spraying with distilled water was considered as the control treatment in three replicates. The seedlings were sprayed with the treatments after reaching the 6–8 leaf stage. Each of the nine treatment solutions was applied to plants in designated plots at a total volume of approximately 3 L (150 mL per/plant). A comprehensive assessment of physiological and biochemical traits was conducted on the third leaf of plants.

### 2.1 Measurement of plant pigments

Before chemical analysis, 0.5 g of fresh plant material was crushed using liquid nitrogen with a mortar and pestle. Twenty milliliters of 80% acetone was added to the sample and added into a centrifuge (6000 rpm [revolutions per minute]) for 10 min. The supernatant was separated and transferred to a flask. A portion of the sample inside the flask was separated for analysis by the spectrophotometer and the absorption value was read at 663 nm for chlorophyll a, 645 nm for chlorophyll b, and 470 nm for carotenoids. Finally, using the following formulas, the amounts of chlorophyll a, b, and carotenoids were calculated as milligrams (mg) per gram of fresh sample weight (Arnon, 1967).

$$\text{Chlorophyll}\;a=\frac{\left(19.3\times A663-0.86\times A645\right)\;V}{100\;W}$$

$$\text{Chlorophyll}\;b=\frac{\left(19.3\times A645-3.6\times A663\right)\;V}{100\;W}$$

$$\text{Carotenoids}=\frac{100\;\left(A470\right)-3.27\;\left(\mathrm{mg}\;\mathrm{chl}.a\right)-104\;\left(\mathrm{mg}\;\mathrm{chl}.b\right)}{227}$$

*V* = the volume of filtered solution (the supernatant from the centrifuge); A = absorption values at 663, 645, and 470 nm; *W* = fresh sample weight (g).

### 2.2 Total phenol content

After completing the extraction process, the extracts were filtered with a rotary evaporator at 40°C and then concentrated with a freeze‐dryer. The phenolic components in the Galega extract were determined by colorimetry using the Folin–Ciocalteu method (Singleton et al., 1999). Following McDonald et al. (2001), 0.5 mL of the Galega extract was mixed with 5 mL of the Folin–Ciocalteu reagent, which was diluted 10 times with distilled water and mixed with 4 mL of 1 M sodium carbonate solution. The mixture was maintained at room temperature for 15 min. The absorption value of the solution was read by a spectrophotometer at 765 nm (Oroojalian et al., 2010). The colorimetric method (Folin–Ciocalteu) was also performed on standard solutions of tannic acid at different concentrations. The standard curve was illustrated against tannic acid absorption (*Y* = 0.00114*X* + 0.01062, where *Y* is the absorption number and *X* is the concentration in parts per million [ppm]). To determine the phenol concentration of the samples, the absorption values of the spectrophotometer were entered in the above equation (*Y*) and the concentration of phenolic components (*X*) was calculated as ppm in the samples.

### 2.3 Determining total flavonoids

Aluminum chloride colorimetric technique was used for determining flavonoids content. Plant methanolic extracts (0.5 mL of 1:10 g/mL) were mixed with 1.5 mL of methanol, 0.1 mL of aluminum chloride (10%), 0.1 mL potassium acetate (1 M), and 2.8 mL of distilled water. The solutions were placed at room temperature for 30 min. The absorbance of each component was measured at 415 nm with a spectrophotometer. The standard curve was made with a methanolic quercetin solution (Sigma Chemical Co.) at concentrations of 250–1000 μg/mL and the curve was illustrated in Microsoft Excel. The linear equation of *y* = *bx* + *a* was obtained from the illustration. After using the spectrophotometer, the absorbance value of each sample was placed as *y*. The *x* variable was the concentration of quercetin (Chang et al., 2002).

### 2.4 Measurement of antioxidant activity

The DPPH (2,2‐Diphenyl‐1‐Picrylhydrazyl) free radical was used for the assessment of antioxidant activity. First, plant extracts were prepared in different concentrations of pure methanol, ranging from 5 × 10^−2^ mg/100 to 5 × 10^−6^. A mixture of DPPH solution (8 mg/100 mg) and plant extracts (1:1 ratio) was made in different concentrations. The absorbance of each sample was measured at 517 nm after 30 min (25°C). The DPPH free‐radical inhibition percentage of each sample was obtained using the following equation.

$$R\%=\frac{\;\mathrm{AD}–\mathrm{AS}}{\mathrm{AD}}\times 100$$

*R*% = inhibition percentage; AD: absorbance of DPPH at 517 nm; AS: absorbance of samples at 517 nm.

To compare the antioxidant activity of the extracts, the IC50 value was used. The IC50 value is the concentration of the extract that inhibits 50% of free radicals (Sun et al., 2007).

### 2.5 Evaluation of antidiabetic properties of Galega extract

The essential components of Galega were extracted by maceration. Galega plants were dried in the shade and ground into powder using a mill. For each extraction, 50 g of plant powder was weighed and poured into Erlenmeyer flasks. Then, the powder was added to ethanol hydroalcohol (80%) in the flasks at a ratio of 8:1. The flasks were placed on a shaker for 48–72 h and then the contents were filtered using a Buchner funnel and Whatman No. 1 filter paper. Again, 75% ethanol was added to the remaining solution before placing it on the shaker for 12 h. Finally, the solution was filtered using a filter paper before rotating in a vacuum distillation apparatus (90 rpm) to make a concentrated extract at 50°C. The concentrated extract was placed in an oven at 48°C for 24 h and the dry Galega powder was obtained (Assadi et al., 2018).

In this experimental study, a total of 48 male NMRI mice (obtained from the Animal House of Urmia University of Medical Sciences, Iran) weighing 20–30 g were used. The animals were kept in separate cages (the cage made of polycarbonate made by Razi Rad Industries) at 23 ± 2°C, proper ventilation, and a 12‐h light/12‐h dark cycle. During this period, commercially available water and food (in the form of a commercial pellet) were provided to them, and each animal was used only once. All experiments were carried out during the light period from 8:00 a.m. to 12:00 p.m.

In the present study, all experiments were carried out in accordance with the Guide for the Care and Use of Laboratory Animals and the Declaration of Tehran University of Medical Sciences as well as the guidelines of National Institutes of Health Guide for the Care and Use of Laboratory Animals (NIH Publications No. 8023, revised 1978). The animals were randomly divided into 13 groups of 6. Before starting the experiments, the mice were adapted to experimental conditions for 7 days. The mice were categorized into four different treatment groups, including the positive control (with diabetes), negative control (without diabetes), metformin, and Galega extract (50 mg/kg) (Abtahi‐Evari et al., 2017; Nagalievska et al., 2018; Pashazadeh et al., 2015; Sabeva et al., 2004).

Alloxan monohydrate (120 mg/kg) (Sigma) was used through intraperitoneal injection to induce diabetes in the positive control and experimental groups (Azizi, 2001; Khademi et al., 2013). To confirm diabetes in the mice, 48 h after the injection, their blood sugar was measured using a diagnostic kit (Pars Azmoun Co.) by the glucose oxidase‐based technique. Mice with blood glucose levels of over 200 mg/dL were considered diabetic. Metformin was administered as 500 mg tablets (Shimi Daro Co.). The positive control and experimental groups received the chemicals by intraperitoneal injection for 10 days. Ultimately, 48 h after the final injection, blood glucose level was measured in all groups (Assadi et al., 2018).

### 2.6 Blood glucose measurement

The glucose index was measured with a diagnostic kit (Pars Azmoun Co.) (Assadi et al., 2018).

### 2.7 Data analysis

Statistical analysis was performed using SAS software (version 9.1). Mean values were compared using Duncan's Multiple Range Test. Data were recorded and summarized in Microsoft Excel and Word.

## 3 RESULTS AND DISCUSSION

### 3.1 Chlorophyll a

**FIGURE 1:**
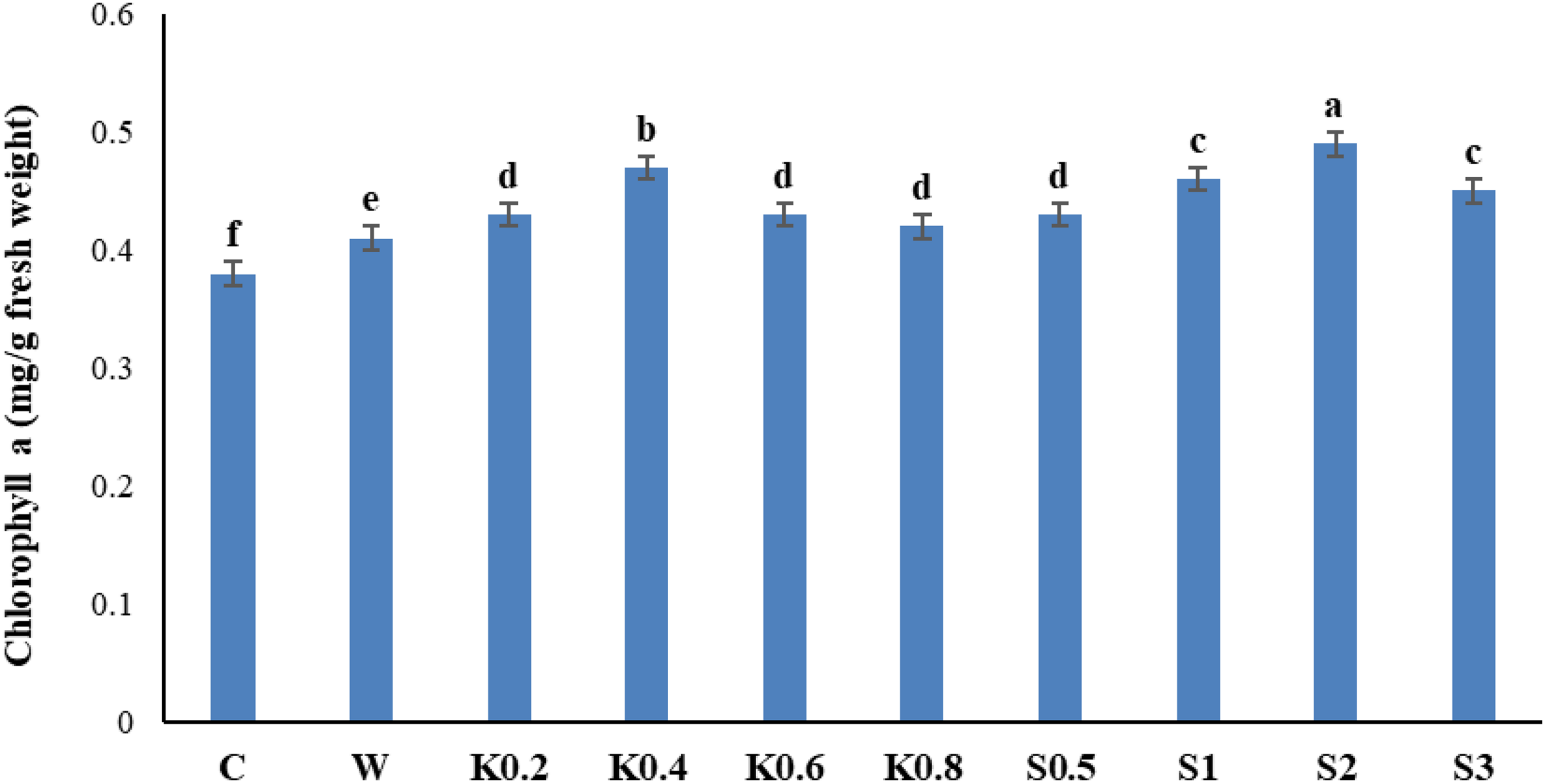
Comparative effects of salicylic acid and chitosan foliar spraying on the amount of chlorophyll a in Galega leaf samples. Means followed by similar letter over each column are not significantly different at 5% level based on the least significant difference (LSD) test. C: control (no spraying), W: distilled water, K0.2: 0.2 g/L chitosan, K0.4: 0.4 g/L chitosan, K0.6: 0.6 g/L chitosan, K0.8: 0.8 g/L chitosan, S0.5: 0.5 mM salicylic acid, S1: one mM salicylic acid, S2: two mM salicylic acid, and S3: three mM salicylic acid.

The amount of chlorophyll a in Galega leaf samples was significantly affected by the foliar spraying of chitosan and salicylic acid (*p* ≤ .01) (Table 1). The comparison of mean values indicated that the amount of chlorophyll a increased by 28.95% in response to the 2 mM salicylic acid treatment, compared to the control treatment (Figure 1).

**TABLE 1:** The results of analysis of variance of the effect of foliar spraying with chitosan and salicylic acid on the leaf pigments of *Galega*.

Source of variation	Degree of freedom	Mean of square		
		Chlorophyll a	Chlorophyll b	Carotenoid
Block	2	0.000003^ns^	0.000003^ns^	0.000001^ns^
Treatment	9	0.003**	0.0032**	0.0003**
Error	18	0.00004	0.00006	0.00003
Coeff variation (%)	‐	1.39	1.48	20.27

** and ns, respectively, a significant difference at the probability level of 1% and the absence of a significant difference.

### 3.2 Chlorophyll b

**FIGURE 2:**
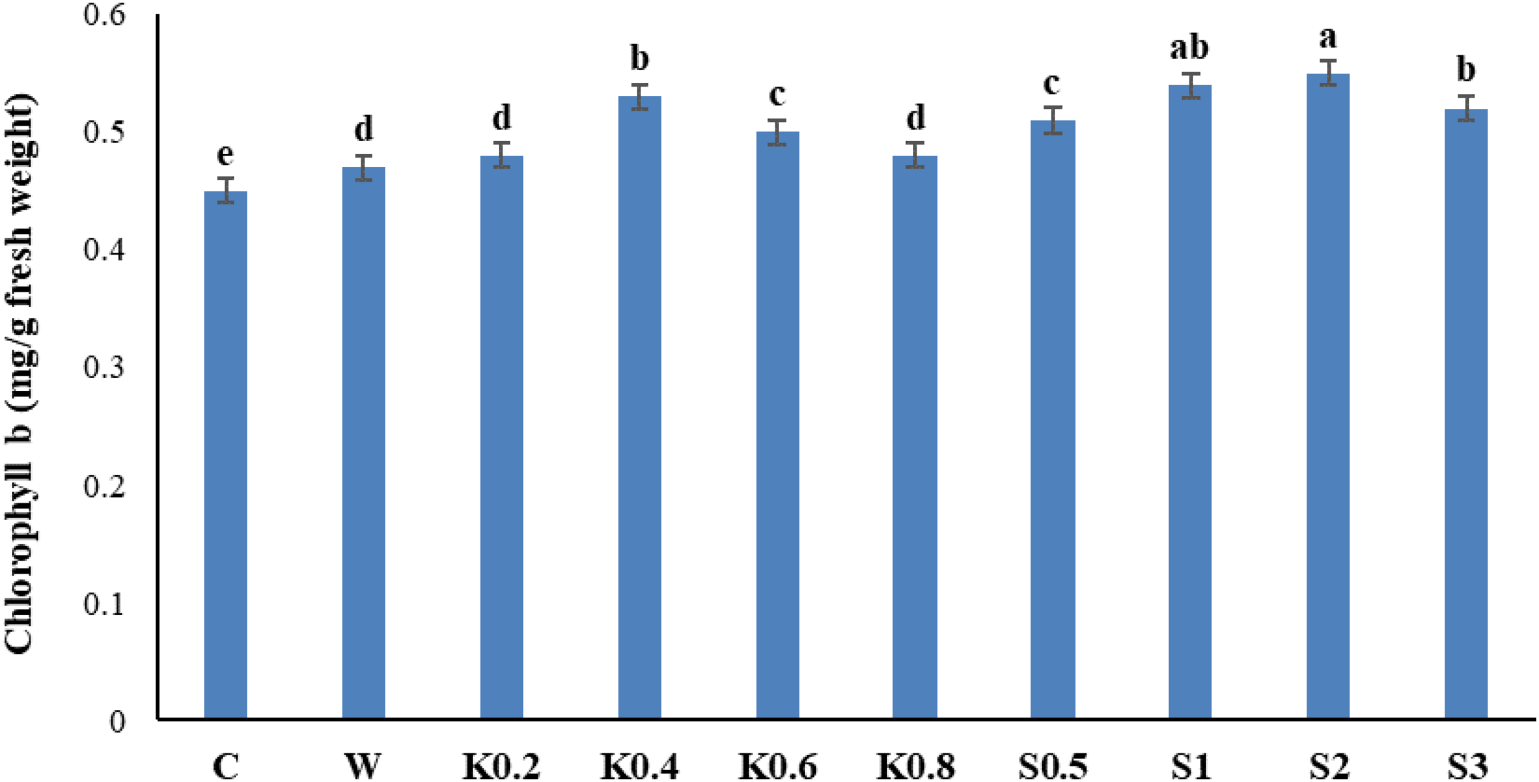
Comparative effects of salicylic acid and chitosan foliar spraying on the amount of chlorophyll b in Galega leaf samples. Means followed by similar letter over each column are not significantly different at 5% level based on the least significant difference (LSD) test. C: control (no spraying), W: distilled water, K0.2: 0.2 g/L chitosan, K0.4: 0.4 g/L chitosan, K0.6: 0.6 g/L chitosan, K0.8: 0.8 g/L chitosan, S0.5: 0.5 mM salicylic acid, S1: one mM salicylic acid, S2: two mM salicylic acid, and S3: three mM salicylic acid.

The amount of chlorophyll b in Galega leaf samples changed significantly under the effect of chitosan and salicylic acid as foliar spraying treatments (*p* ≤ .01) (Table 1). The comparison of mean values showed that leaf samples of the 2 mM salicylic acid treatment had more chlorophyll b (0.55 mg/g FW) than the other treatment groups. However, the observed value of this treatment group was not significantly different from the effect of 1 mM salicylic acid on chlorophyll b (0.54 mg/g FW). The lowest amount of chlorophyll b (0.45 mg/g FW) was observed in leaf samples with no foliar treatment (control). Intermediate concentrations of salicylic acid and chitosan caused higher amounts of chlorophyll b in the leaf samples, compared to the effects of low or high concentrations of chitosan and salicylic acid (Figure 2).

Salicylic acid is reportedly known for its effective increase in the production of plant hormones, including auxins and cytokinins. These hormones improve plant growth, create more plant pigments, increase photosynthesis, and, thus, enhance total yield (Shakirova et al., 2003). In line with the findings of the current research, it was reported that the foliar application of salicylic acid at a low concentration (1 mM) benefited the photosystem under nonstress conditions (Moustakas et al., 2022). Although the effects of exogenously applied salicylic acid are reportedly variable, stress or nonstress conditions are significantly effective in determining the ultimate outcome of using salicylic acid (Janda et al., 2014). Since nonstress conditions were used in the current research, a small concentration of salicylic acid (2 mM) was suitable for increasing the photosynthetic activity.

Chitosan is known for its proven effects on improving chlorophyll content in sweet pepper plants (ALKahtani et al., 2020), barley (Hafez et al., 2020), stevia (Gerami et al., 2020), and *Prunus davidiana* (Xu et al., 2020). The combination of exogenous chitosan and salicylic acid has been reportedly useful for improving phytochemical parameters in milk thistle plants (Ghanbari Moheb Seraj et al., 2021). Since chlorophyll has a positive role in photosynthesis and its related enzymes, a larger availability of chlorophyll content in the leaf can improve energy production in plants and cause a more efficient transfer of photo‐assimilates from source to reservoir (Keshavarz & Modarres Sanavy, 2015).

### 3.3 Carotenoids

**FIGURE 3:**
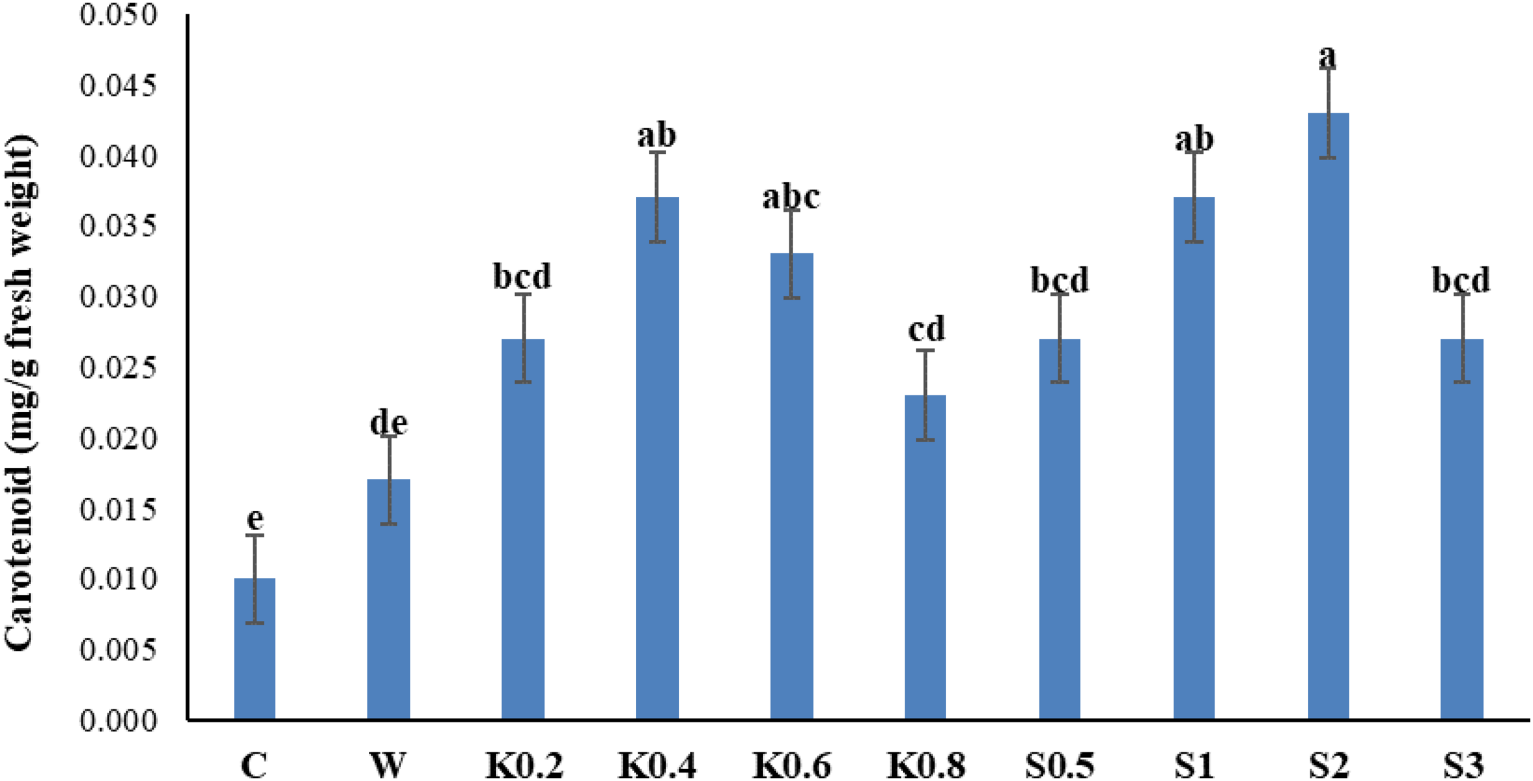
Comparative effects of salicylic acid and chitosan on the carotenoid content of Galega plant leaves. Means followed by similar letter over each column are not significantly different at 5% level based on the least significant difference (LSD) test. C: control (no spraying), W: distilled water, K0.2: 0.2 g/L chitosan, K0.4: 0.4 g/L chitosan, K0.6: 0.6 g/L chitosan, K0.8: 0.8 g/L chitosan, S0.5: 0.5 mM salicylic acid, S1: one mM salicylic acid, S2: two mM salicylic acid, and S3: three mM salicylic acid.

The foliar treatments of chitosan and salicylic acid significantly improved the amount of carotenoids in Galega leaf samples (*p* ≤ .01) (Table 1). The highest amount of carotenoids developed in response to the 2 mM salicylic acid treatment, which was not significantly different from the effect of 1 mM salicylic acid. The lowest amount of carotenoids was observed in the control treatment (Figure 3).

By reducing the amount of oxidative stress and increasing the amount of proline, salicylic acid can protect cell membranes and organelles from damage. Salicylic acid reportedly reduced the rate of lipid peroxidation and increased the antioxidant capacity of red bean plants (Mahdavian, 2022). The protective effect of chitosan is usually extended to cellular protein production and the structure of proteins and enzymes, thereby preventing their oxidation and decomposition (Lopez‐Moya et al., 2019; Shariatinia, 2019). Spraying salicylic acid on plants increased the carotenoid content, and, thus, improved the antioxidant capacity to reduce the rate of lipid peroxidation and hydrogen peroxide (H_2_O_2_) production, which is in agreement with previous research on milk thistle (Ghanbari Moheb Seraj et al., 2021). These effects can protect plant cells, photosynthetic membranes, and photosynthetic pigments from chlorophyll catabolism (Costa et al., 2005). In the juice sacs of satsuma mandarin, exogenous salicylic acid contributed to changing the expression of the *CitWRKY70* gene and led to a greater biosynthesis of flavonoids and carotenoids (Yamamoto et al., 2020). The application of chitosan on *Pinus pinaster* caused an increase in the production of carotenoids, among many other variables, by increasing the expression level of the *DEF* gene (Nunes da Silva et al., 2021).

### 3.4 Total phenol content

**FIGURE 4:**
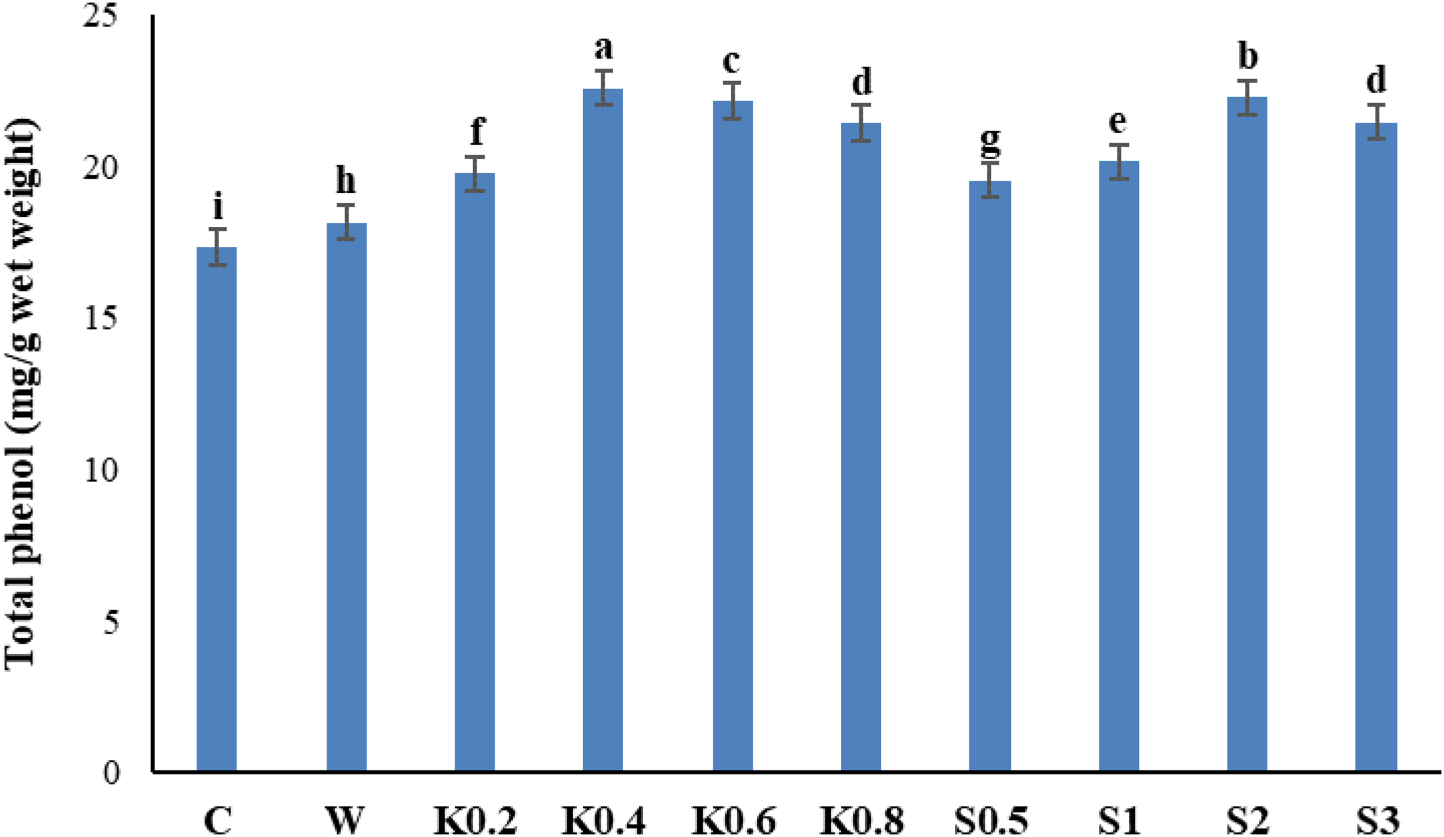
Comparative effects of salicylic acid and chitosan on the total phenol content of Galega leaf samples. Means followed by similar letter over each column are not significantly different at 5% level based on the least significant difference (LSD) test. C: control (no spraying), W: distilled water, K0.2: 0.2 g/L chitosan, K0.4: 0.4 g/L chitosan, K0.6: 0.6 g/L chitosan, K0.8: 0.8 g/L chitosan, S0.5: 0.5 mM salicylic acid, S1: one mM salicylic acid, S2: two mM salicylic acid, and S3: three mM salicylic acid.

The amount of total phenol in Galega leaf samples increased significantly because of the foliar application of chitosan and salicylic acid (*p* ≤ .01) (Table 2). The total phenol content increased by 30.31% by application of 0.4 g/L chitosan compared to the control (Figure 4). The application of salicylic acid (2 mM) was significantly effective in increasing the total phenol content (22.2 mg/g FW) compared to the control group, with no foliar application (17.32 mg/g FW) and with distilled‐water application (18.15 mg/g FW).

**TABLE 2:** The results of analysis of variance of the effect of foliar spraying with chitosan and salicylic acid on some chemical compounds of *Galega*.

Source of variation	Degree of freedom	Mean of square			
		Total phenol	Total flavonoid	Antioxidant activity	Total alkaloid
Block	2	0.005^ns^	0.37*	0.075^ns^	0.000003^ns^
Treatment	9	9.70**	182.90**	66.53**	0.00003**
Error	18	0.0027	0.114	0.07	0.0038
Coeff variation (%)	‐	0.25	1.21	0.38	3.57

**, * and ns, respectively, a significant difference at the probability level of 1%, 5% and the absence of a significant difference.

In basil, chitosan increased the total phenol content and terpenic compounds, especially rosmarinic acid and eugenol (Kim & Hwang, 2014). In a relevant case of research, chitosan (150 mg/L) was applied onto the root hairs of *Artemisia annua* L., which increased the amount of artemisinin to 1.8 μg/mg (Putalun et al., 2007). Chitosan induces the production of phytoalexins (Righetti et al., 2007) as well as many phenolic and terpenoid compounds (Kim et al., 2005). According to recent studies, chitosan increased the amount of phenolic compounds in white flax (*Linum album*) (Esmaeilzadeh Bahabadi et al., 2013), *Pinus pinaster* (Nunes da Silva et al., 2021), and eggplant (Mandal, 2010), which confirm the current results.

Elicitors such as chitosan may activate new genes that facilitate enzymatic activity and induce significant outcomes through biosynthetic pathways to increase the production of secondary metabolites (Mehregan et al., 2017). Increasing the exogenous level of chitosan up to 100 mg/L increased the amount of total phenol in *Dracocephaltum kotschyi* (Ayyobi et al., 2017). The consumption of chitosan in oregano plants increased the amount of 12 polyphenols (i.e., 4 phenolic acids and 8 flavonoids). The increase in polyphenol content resulted from the stimulatory effect of chitosan on biosynthetic enzymes, such as phenylalanine ammonialyase (PAL) and polyphenol chalcone synthase (Malekpoor et al., 2017).

Salicylic acid is known as a key messenger component in the activation of plant defense responses. The variety of responses in plant interactions with the environment leads to the biosynthesis and accumulation of various secondary metabolites, including plant phenolic compounds (Herrera‐Vásquez et al., 2015). Phenols are potent antioxidant compounds in plant tissues that play important roles due to their skeletal structure, the most important of which is to eliminate oxygen‐free radicals produced in stressful conditions. Accordingly, phenols protect cytoplasmic and chloroplastic structures from the negative effects of stress and prevent the oxidation of lipids by inhibiting lipoxygenase activity (El‐Taher et al., 2021). The DPPH radical is a free, stable, organic, and nitrogenous compound that is widely used for assessing free‐radical scavenging activity. The DPPH scavenging test is commonly used for determining the antioxidant ability of compounds to scavenge free radicals (Baliyan et al., 2022). In plants, there is a positive correlation between phenolic compounds and the scavenging of free radicals, meaning that plants become more capable of producing secondary metabolites when free radicals are scavenged more effectively by an abundance of phenolic compounds (Kozłowska et al., 2021).

### 3.5 Total flavonoid content

**FIGURE 5:**
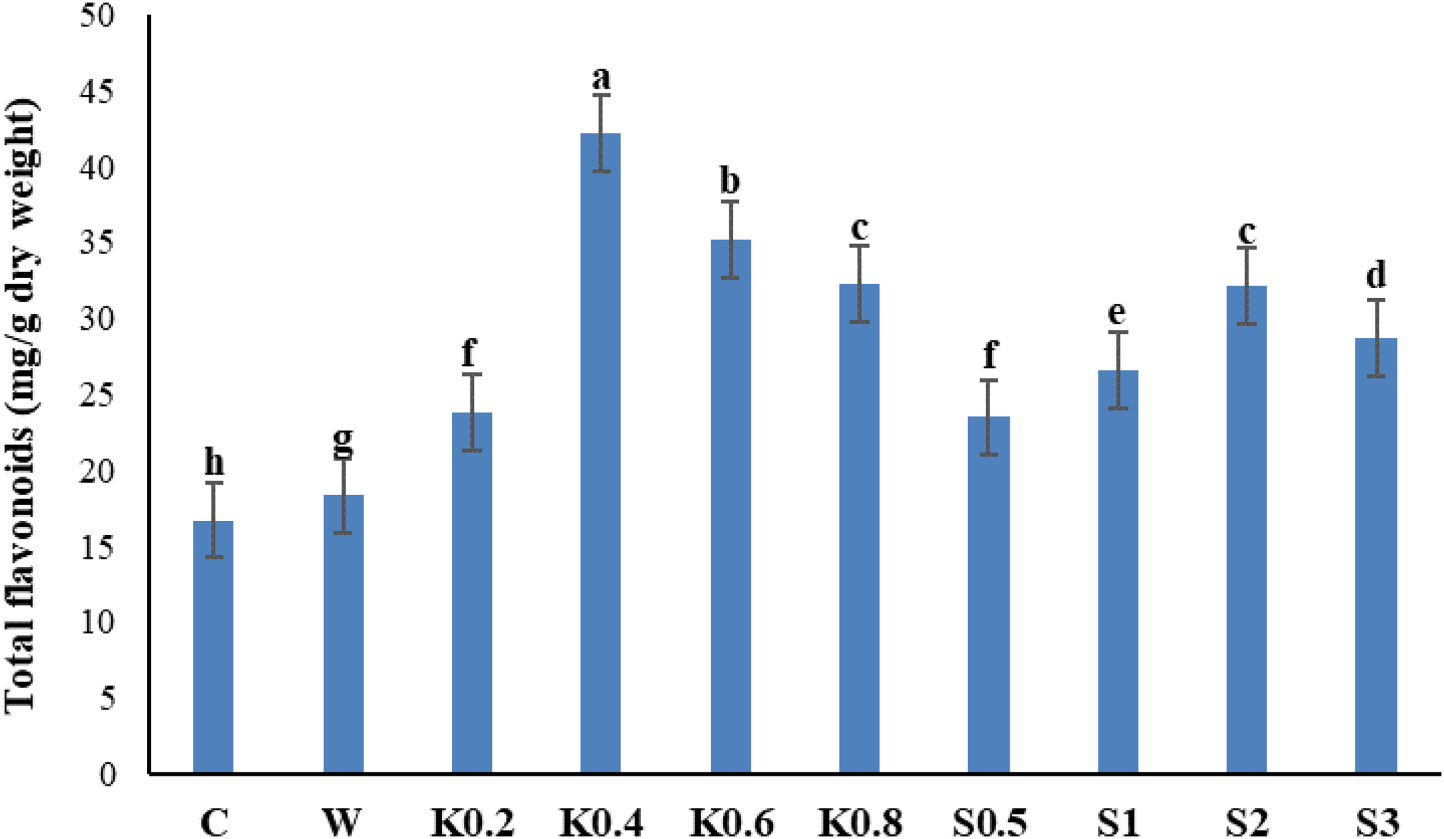
Comparative effects of salicylic acid and chitosan foliar spraying on the total flavonoid content of Galega leaves. Means followed by similar letter over each column are not significantly different at 5% level based on the least significant difference (LSD) test. C: control (no spraying), W: distilled water, K0.2: 0.2 g/L chitosan, K0.4: 0.4 g/L chitosan, K0.6: 0.6 g/L chitosan, K0.8: 0.8 g/L chitosan, S0.5: 0.5 mM salicylic acid, S1: one mM salicylic acid, S2: two mM salicylic acid, and S3: three mM salicylic acid.

The amount of total flavonoids increased significantly by the application of chitosan and salicylic acid as foliar sprays (*p* ≤ .01) (Table 2). Among the treatment groups, the amount of total flavonoids increased most significantly (42.16 mg/g FW) by the application of 0.4 g/L chitosan, whereas the lowest amount of total flavonoids (16.72 mg/g FW) was observed in the leaves of Galega plants which received no foliar treatment (Figure 5). The application of 2 mM salicylic acid was most effective in increasing the total flavonoid content (32.14 mg/g FW), compared to the other salicylic acid treatments.

The phenylpropanoid pathway is one of the main sources of secondary metabolite production in plants, which ultimately contributes to the production of various types of compounds, such as anthocyanins, flavonoids, ultraviolet (UV) protectors, antimicrobial furanocoumarins, isoflavonoids, phytoalexins, lignins, and phenolic esters (Fraser & Chapple, 2011). Plants produce phenylpropanoid compounds in response to biotic stress caused by fungi and bacteria. These compounds are largely responsible for creating defense mechanisms in plants (König et al., 2014). Salicylic acid is one of those important components that improve plant defense systems and is considered a prominent signaling molecule for defense in plants. In the initial stages of the phenylpropanoid pathway, as one of the two pathways of its biosynthesis, salicylic acid is biosynthesized from cinnamate, as a result of PAL enzyme activity, and activates the systemic acquired resistance (SAR) pathway to induce systemic acquired resistance in plants (Seyfferth & Tsuda, 2014). Phenylalanine ammonialyase is the main enzyme of the phenylpropanoid pathway. It catalyzes the first stage of phenylpropanoid compound synthesis and acts through a nonoxidative deamination of phenylalanine to convert into ammonia and trans‐cinnamic acid (Kim & Hwang, 2014). PAL activity is induced during plant–pathogen interactions and environmental stress (Uppalapati et al., 2007).

As an extracellular biostimulant, chitosan affects enzymes that are involved in the phenylpropanoid pathway and the production of secondary metabolites (Chakraborty et al., 2009; Korsangruang et al., 2010). Previous research showed that using chitosan as a cellular stimulant of secondary metabolite biosynthesis can effectively improve the phytochemical profile of various plant species (Cai et al., 2012; Kim, 2005; Mhlongo et al., 2016; Udomsuk et al., 2011; Wiktorowska et al., 2010). However, the concentration of the biochemical stimulus, depending on the plant species, is an important parameter in determining the extent of stimulation and the intensity of the plant's response to the stimulus (Vasconsuelo & Boland, 2007).

### 3.6 Antioxidant activity

**FIGURE 6:**
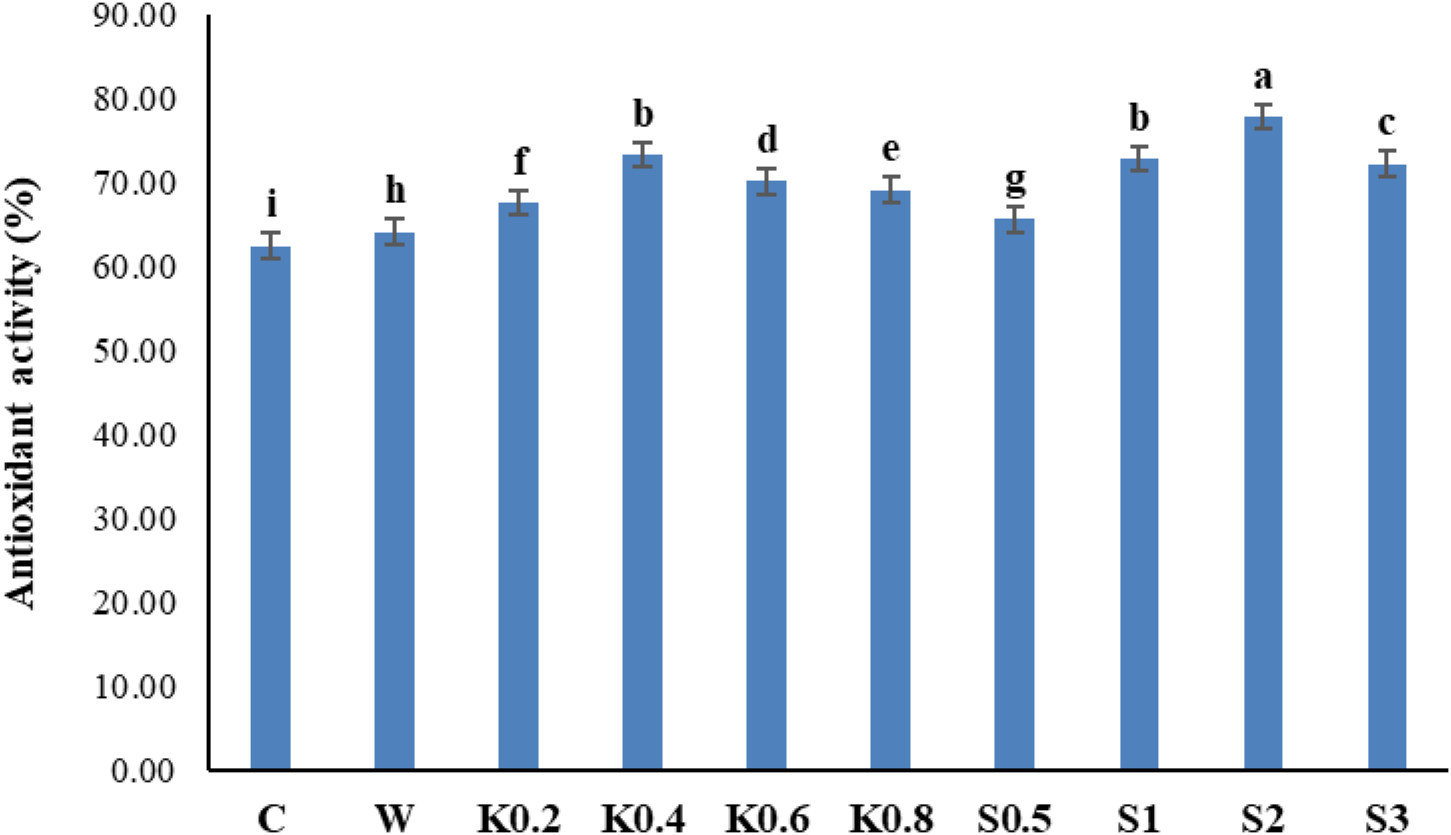
Comparison of the average effect of foliar spraying of salicylic acid and chitosan on the antioxidant activity of Galega medicinal plant. Means followed by similar letter over each column are not significantly different at 5% level based on the least significant difference (LSD) test. C: control (no spraying), W: distilled water, K0.2: 0.2 g/L chitosan, K0.4: 0.4 g/L chitosan, K0.6: 0.6 g/L chitosan, K0.8: 0.8 g/L chitosan, S0.5: 0.5 mM salicylic acid, S1: one mM salicylic acid, S2: two mM salicylic acid, and S3: three mM salicylic acid.

The antioxidant activity of Galega leaf samples increased significantly by the foliar application of chitosan and salicylic acid (*p* ≤ .01) (Table 2). The highest antioxidant activity (77.72%) was observed in samples treated with 2 mM salicylic acid. Also, treatments of 1 mM salicylic acid and 0.4 g/L chitosan caused significantly high levels of antioxidant activity (72.73% and 73.18%, respectively), which were not significantly different from the 2 mM salicylic acid treatment. The lowest amount of antioxidant activity (62.35%) was observed in samples of the control, without any foliar application (Figure 6).

Salicylic acid (SA) is an important signaling molecule in plants and induces physiological responses to various biotic and abiotic stresses. The application of exogenous salicylic acid has reportedly alleviated the toxic effects of heavy metals, such as cadmium, lead, and mercury (Sharma et al., 2020). Salicylic acid plays an important role in regulating a number of physiological processes in plants, such as the regulation of growth and development, ion absorption and transport, and membrane permeability (Saleem et al., 2021). In addition, it affects the production rate of reactive oxygen species (ROS) under stress conditions and changes the activity of antioxidant enzymes, such as catalase, peroxide, and superoxide dismutase, thereby making plants more tolerant to stress and increasing their performance in nonstress conditions as well (Moustakas et al., 2022). Salicylic acid is an important signaling molecule for mediating plant responses to environmental stresses (Raskin, 1992).

Several studies have shown that the main components of the cell wall of many fungal species, such as chitosan, increase the amount of secondary metabolites (Kang et al., 2004; Pu et al., 2009). The antioxidant activity of chitosan is carried out by different mechanisms (Park et al., 2004). It increases the activity of antioxidant enzymes and facilitates the eradication of free radicals OH and O^−2^ to protect DNA (Harish Prashanth et al., 2007; Kim & Thomas, 2007). According to relevant research, exogenous chitosan reportedly increased the activity of antioxidant enzymes in grapes (Aazami et al., 2023). Also, the activity of catalase and polyphenol oxidase, as antioxidant enzymes, increased in eggplant roots when treated with chitosan (Mandal, 2010). The activity of peroxidase and catalase increased in safflower and corn seedlings by the effect of chitosan (Guan et al., 2009; Mahdavi et al., 2012). The catalase enzyme actively functions in the peroxisome, cytosol, and mitochondria to convert H_2_O_2_ into H_2_O and O_2_, while ascorbate peroxidase uses ascorbate as an electron donor at the beginning of the ascorbate glutathione cycle and plays an important role in the detoxification of H_2_O_2_ in cells (Mckersie & Leshem, 1994). Recently, chitosan has attracted a lot of attention because of its ability to increase antioxidant activity in plants against biotic stress (Nunes da Silva et al., 2021) and abiotic stress (Hidangmayum et al., 2019). Several studies have shown that chitosan acts as a bio‐elicitor and may have the potential to scavenge free radicals (Kim & Thomas, 2007; Yen et al., 2008). The antioxidant activity of chitosan is fundamentally described by several mechanisms (Park et al., 2004). In *Mentha piperita*, higher levels of chitosan application caused an increase in the amount of total phenolic and flavonoid compounds, which ultimately increased the antioxidant activity of plant extracts (Salimgandomi & Shabrangi, 2016).

### 3.7 Total alkaloid content

**FIGURE 7:**
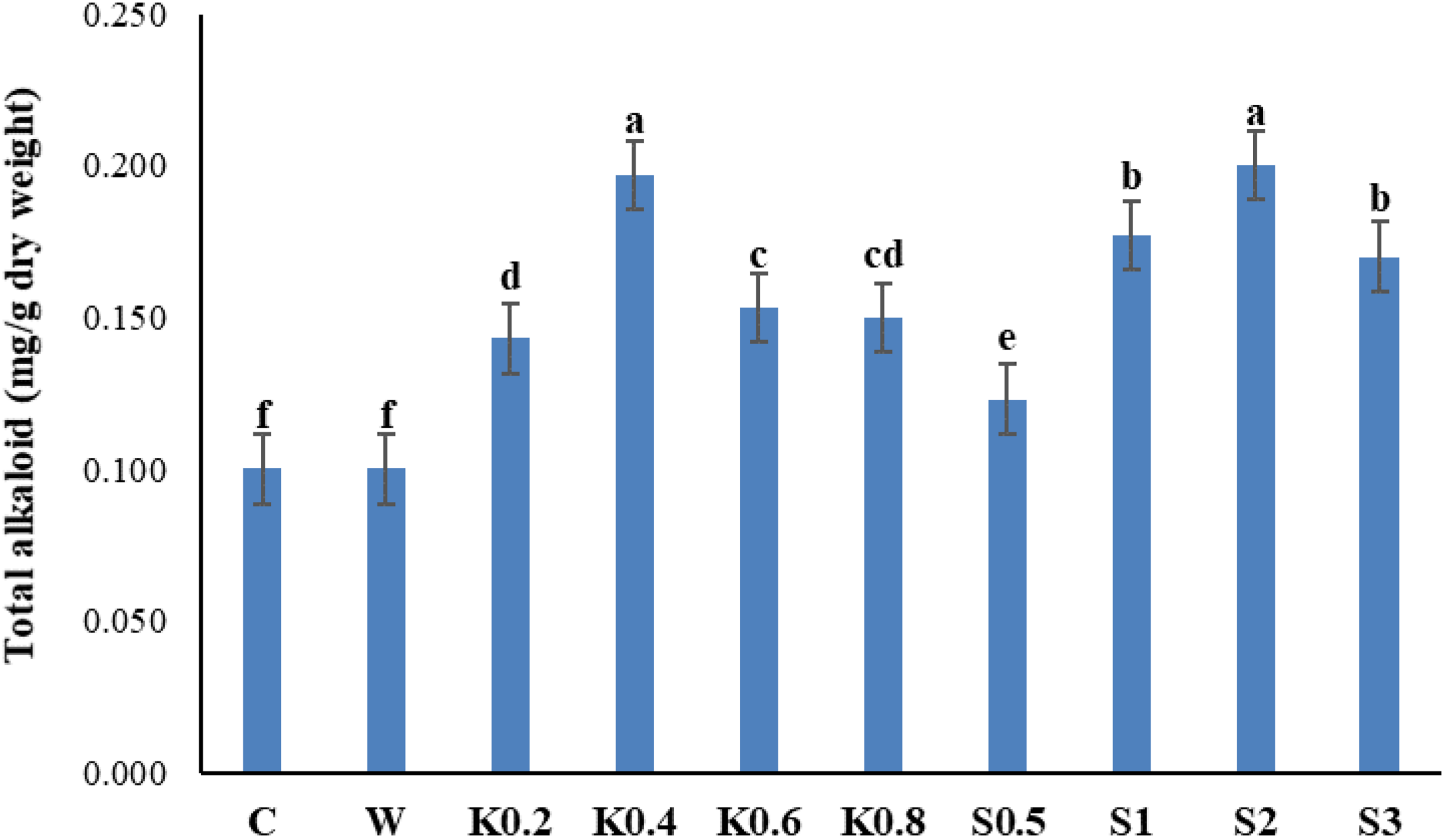
Comparative effect of salicylic acid and chitosan foliar spraying on the total alkaloid content of Galega leaf samples. Means followed by similar letter over each column are not significantly different at 5% level based on the least significant difference (LSD) test. C: control (no spraying), W: distilled water, K0.2: 0.2 g/L chitosan, K0.4: 0.4 g/L chitosan, K0.6: 0.6 g/L chitosan, K0.8: 0.8 g/L chitosan, S0.5: 0.5 mM salicylic acid, S1: one mM salicylic acid, S2: two mM salicylic acid, and S3: three mM salicylic acid.

The amount of total alkaloids in Galega leaf samples improved significantly by the effect of foliar spraying with chitosan and salicylic acid (*p* ≤ .01) (Table 2). Plants treated with 2 mM salicylic acid accumulated the highest amount of alkaloids in their leaves (0.2 mg/g DW). This had a slight difference compared to the effect of 0.4 g/L chitosan, which also caused a high amount of alkaloid content (0.197 mg/g DW). In the control treatment, without any foliar application, the lowest level of alkaloid content was observed (0.1 mg/g DW), which was similar to the effect of distilled water (0.1 mg/g DW) (Figure 7).

Chitin and chitosan are fungal elicitors with major chemical compounds in the cell wall of many fungal species. When applied exogenously, their functional role is to increase the amount of secondary metabolites in plants, especially those involved in plant defense mechanisms (Pu et al., 2009). In a relevant study, the use of chitosan as a biological elicitor on common rue plants (*Ruta graveolens*) caused a significant increase in the amount of coumarin compounds and alkaloid compounds (Orlita et al., 2008). Also, the amount of alkaloid production increased in periwinkle plants when chitosan was applied in association with endophyte microbes (Farouk et al., 2022). In agreement with the current results, chitosan served as a biostimulant for the growth of different medicinal plants, thereby increasing their vegetative growth, performance, and secondary metabolite production (Mehregan et al., 2017; Ozhan et al., 2017; Salehi & Rezayatmand, 2017). Several studies have shown that salicylic acid is effective in stimulating the production of many secondary metabolites, such as terpenoids, coumarin derivatives, alkaloids, and flavonoids (Ismailzadeh Bahabadi & Rezaei‐Nodehi, 2014). Salicylic acid reportedly improved the production of alkaloids in woad (*Isatis tinctoria*) via the elicitation of hairy roots (Gai et al., 2019).

### 3.8 Diabetes

**FIGURE 8:**
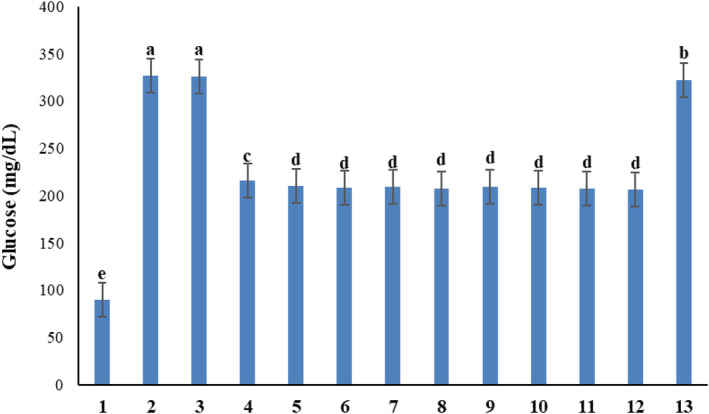
Comparative effects of metformin and Galega plant extracts on glucose levels in laboratory mice. Means followed by similar letter over each column are not significantly different at 5% level based on the least significant difference (LSD) test. 1: Nondiabetic mice that received no injection; 2: Diabetic mice that received no injection; 3: Dimethylsulfoxide (DMSO); 4: Diabetic mice with metformin injection; 5: Diabetic mice with the injection of chitosan extract 0.2 g/L; 6: Diabetic mice with the injection of chitosan extract 0.4 g/L; 7: Diabetic mice with the injection of chitosan extract 0.6 g/L; 8: Diabetic mice with the injection of chitosan extract 0.8 g/L; 9: Diabetic mice with the injection of 0.5 mM salicylic acid extract; 10: Diabetic mice with the injection of 1 mM salicylic acid extract; 11: Diabetic mice with the injection of 2 mM salicylic acid extract; 12: Diabetic mice with the injection of 3 mM salicylic acid extract; and 13: Diabetic mice with the injection of a placebo solvent.

The effects of chitosan and salicylic acid on plants changed their phytochemical profile. After obtaining extracts from Galega leaves, the changes in phytochemical profile were observed in the therapeutic functionality of the Galega extract in reducing the blood glucose level of laboratory mice. Administering extracts of a stronger phytochemical profile resulting from the foliar applications caused a significant decrease in blood glucose level (*p* ≤ .01) (Table 3). The most effective extract resulted from plants that had received the foliar application of 3 mM salicylic acid, leading to a blood glucose level of 205.99 mg/dL, compared to the control group of diabetic mice that did not receive any treatment and had a blood glucose level of 325.89 mg/dL. The least effective extract resulted from plants that had been treated with 0.2 g/L chitosan and led to a glucose level of 209.75 mg/dL, which was not significantly different from the most effective extract that caused the lowest glucose level (205.99 mg/dL) in diabetic mice. Administering metformin did not reduce the glucose level as effectively as the Galega extracts. Metformin reduced the glucose level to 215.6 mg/dL, which was not in the same statistical group of glucose levels reduced by Galega extracts (Figure 8).

**TABLE 3:** The results of the analysis of variance of the extract of the test treatments on the glucose level of mice.

Source of variation	Degree of freedom	Mean of square
		Glucose
Treatment	12	12,685.87**
Error	24	4.04
Coeff variation (%)	‐	0.89

** Indicates a significant difference at the probability level of 1%.

For diabetic patients, the consumption of effective medicinal plants can have a significant role in controlling diabetes while having fewer side effects. Although medicinal plants are effective in stimulating insulin secretion, it seems unlikely that the plants can replace insulin completely. These natural sources are effective in the treatment of diabetes by stimulating the biosynthesis and secretion of endogenous insulin, while also enhancing the action of insulin. Therefore, the direct effect of some medicinal plants on the activity of pancreatic cells and insulin secretion can lead to suggestions that the consumption of these medicinal plants can assist patients in their struggle against diabetes. Nonetheless, a safe clinical approach to this issue must be brought into the professional supervision of clinical conduct. It is possible that some medicinal plants have chemical interactions with synthetic drugs, and, thus, the consumption of herbal extracts with drugs requires further trials before clinical approval (Bouyahya et al., 2021; Hamza et al., 2019; Jacob & Narendhirakannan, 2019).

Using chitosan as a phytochemical stimulus has created a promising perspective in the production of phytoalexins and secondary metabolites in biotechnology (Orlita et al., 2008). The biological activity of chitosan is known to emanate from its ability to bind with membrane receptors for inducing a larger production of secondary metabolites, depending on molecular weight and the degree of acetylation (Kauss et al., 1989). The positive effects of chitosan on the production of secondary metabolites have been proven in previous research. The encapsulation of resveratrol–zinc oxide complex with chitosan reportedly alleviated the diabetic status of laboratory mice (Du et al., 2020). Curcumin‐loaded chitosan was effective in reducing blood glucose levels because chitosan nanoparticles increased the bioavailability of curcumin for the treatment of diabetic patients (Rezkita et al., 2020). Chitosan can effectively increase the production of secondary metabolites by affecting the gene expression of key enzymes in the biosynthetic pathway of phenylpropanoid compounds (Kamalipourazad et al., 2017). There is little information in the available literature on the role of salicylic acid in the enrichment of plant extracts while precisely aiming to increase their therapeutic effects against diabetes. Therefore, the current research bears novelty in this respect.

## 4 CONCLUSIONS

In Galega plants, the role of chitosan and salicylic acid as growth stimulants effectively increased the amount of total phenols, total flavonoids, and antioxidant activity, which are interlinked variables in the enrichment of the phytochemical profile of the Galega extract. The amount of alkaloids increased significantly in Galega plants under the effect of chitosan (0.4 g/L) and salicylic acid (2 mM). The novelty of this research was to demonstrate how chitosan and salicylic acid can enrich the phytochemical profile of Galega extracts to increase their effectiveness against diabetes type II. Blood glucose levels decreased significantly in diabetic mice after administering Galega extracts obtained from Galega plants that had been treated with chitosan and salicylic acid. The Galega extracts were significantly more effective than metformin in reducing the blood glucose level. Future research can consider the compatibility and safeness of administering Galega extracts in combination with commercial antidiabetic drugs.

## AUTHOR CONTRIBUTIONS

**Hassan Nourafcan:** Conceptualization (equal); funding acquisition (equal); methodology (equal); project administration (equal); supervision (equal); validation (equal); writing – review and editing (equal). **Farinaz Angouti:** Data curation (equal); formal analysis (equal); investigation (equal); methodology (equal); software (equal); writing – original draft (equal). **Sakineh Saeedi Sar:** Investigation (equal); methodology (equal); supervision (equal); validation (equal); writing – review and editing (equal). **Assad Assadi:** Methodology (equal); resources (equal); supervision (equal); validation (equal); writing – review and editing (equal). **Raheleh Ebrahimi:** Investigation (equal); methodology (equal); supervision (equal); validation (equal).

## CONFLICT OF INTEREST STATEMENT

The authors declare no conflicts of interest.

## Data Availability

The data that support the findings of this study are available from the corresponding author upon reasonable request.
